# Prescription writing pattern among the dental practitioners of a tertiary care hospital in Karachi

**DOI:** 10.1186/s12875-024-02532-z

**Published:** 2024-07-27

**Authors:** Ruqaya Shah, Jehan Alam, Sheheryar Minallah, Maria Shabbir, Maria Shakoor Abbasi, Kashif Aslam, Naseer Ahmed, Artak Heboyan

**Affiliations:** 1 Department of Oral & Maxillofacial Surgery, Jinnah Postgraduate Medical Centre, Karachi, Pakistan; 2 Department of Oral & Maxillofacial Surgery, Shaheed Mohtarma Benazir Bhutto institute of Trauma, Karachi, Pakistan; 3 Department of Prosthodontics, School of Dentistry, Shaheed Zulfiqar Ali Bhutto Medical University, Islamabad, Pakistan; 4 Department of Prosthodontics, Dow Dental College, Dow University of Health Sciences, Karachi, Pakistan; 5 Department of Prosthodontics, Altamash Institute of Dental Medicine, Karachi, 75500 Pakistan; 6 Department of Research Analytics, Saveetha Institute of Medical and Technical Sciences, Saveetha Dental College and Hospitals, Saveetha University, Chennai, 600 077 India; 7 Department of Prosthodontics, Faculty of Stomatology, Yerevan State Medical University after Mkhitar Heratsi, Str. Koryun 2, Yerevan, 0025 Armenia; 8 Department of Prosthodontics, School of Dentistry, Tehran University of Medical Sciences, North Karegar St, Tehran, Iran

**Keywords:** Dental drugs, Drug prescription, Antibiotics prescription, Drug dose, Analgesics prescription

## Abstract

**Objective:**

To identify the frequency and types of prescription errors, assess adherence to WHO prescribing indicators, and highlight the gaps in current prescribing practices of Junior dental practitioners in a tertiary care hospital in Karachi, Pakistan.

**Methods:**

This cross-sectional study was conducted from January 2021 to March 2021. The study included the prescriptions by house surgeons and junior postgraduate medical trainees for walk-in patients visiting the dental outpatient department. A total of 466 prescriptions were evaluated for WHO core drug prescribing indicators. The prescription error parameters were prepared by studying the WHO practical manual on guide to good prescribing and previous studies. Prescription errors, including errors of omission related to the physician and the patients, along with errors of omission related to the drug, were also noted. The statistical analysis was performed with SPSS version 25. Descriptive analysis was performed for qualitative variables in the study.

**Results:**

The average number of drugs per encounter was found to be 3.378 drugs per prescription. The percentage of encounters with antibiotics was 96.99%. Strikingly, only 16.95% of the drugs were prescribed by generic names and 23.55% of drugs belonged to the essential drug list. The majority lacked valuable information related to the prescriber, patient, and drugs. Such as contact details 419 (89.9%), date 261 (56%), medical license number 466 (100%), diagnosis 409 (87.8%), age and address of patient 453 (97.2%), form and route of drug 14 (3%), missing drug strength 69 (14.8%), missing frequency 126 (27%) and duration of treatment 72 (15.4%). Moreover, the wrong drug dosage was prescribed by 89 (19%) prescribers followed by the wrong drug in 52 (11.1%), wrong strength in 43 (9.2%) and wrong form in 9 (1.9%). Out of 1575 medicines prescribed in 466 prescriptions, 426 (27.04%) drug interactions were found and 299 (64%) had illegible handwriting.

**Conclusion:**

The study revealed that the prescription writing practices among junior dental practitioners are below optimum standards. The average number of drugs per encounter was high, with a significant percentage of encounters involving antibiotics. However, a low percentage of drugs were prescribed by generic name and from the essential drug list. Numerous prescription errors, both omissions and commissions, were identified, highlighting the need for improved training and adherence to WHO guidelines on good prescribing practices. Implementing targeted educational programs and stricter regulatory measures could enhance the quality of prescriptions and overall patient safety.

## Introduction

A medical prescription is a legal document used to specify the medications and treatment needed to cure a medical condition [1]. The judicious selection and prescribing of appropriate drugs is important in effectively improving a patient’s health [2]. However, the irrational and immoderate drug prescription not only causes financial burdens, adverse effects and difficulties in obtaining the required medications. Therefore, the necessity for drugs and medications included in prescriptions should be transparent and justified to mitigate potential negative impacts and enhance overall treatment efficacy and safety. [2, 3].

The ability to prescribe commonly used medications safely and effectively is a core competency of a qualified practitioner. The reported frequency of prescription errors varies between 39 and 74% of all medication errors [4, 5]. In developing countries, many people suffer annually due to medication errors resulting from incorrect drug selection, inadequate prescription and misinterpretation [4]. Errors also occur in developed countries, particularly among junior doctors or residents. A meta-analysis showed that junior doctors were responsible for most of the prescription errors in hospitals, varying from 2 to 514 per one thousand prescriptions and from 4.2 to 82% of patients [6]. Furthermore, a study at London University Hospital recorded 135 medical errors per week, of which a quarter were potentially serious, with the most errors from third- or fourth-year house officers [5].

To improve the treatment quality and to avoid medication errors, it is necessary to establish and follow standard healthcare protocols [7]. The World Health Organization (WHO) has developed drug prescription patterns that include indicators to help medical practitioners write appropriate prescriptions and avoid errors [7, 8]. An ideal prescription should comprise all elements: superscription (date, particulars of the prescribing doctor, patient details, and the symbol Rx), inscription (name of the drug, formulation, and unit dosage), subscription (quantity and dosage form for the pharmacists), transcription (directions for the patient), and the prescriber’s signature along with the registration number issued by the medical council [9].

In the field of dentistry, the most commonly prescribed drugs are NSAIDs and antibiotics. Due to the characteristics of these drugs, it is mandatory to determine accurate doses and be aware of any adverse or toxic effects [10, 11]. However, junior and inexperienced practitioners are often prone to making prescription errors, which can lead to adverse drug reactions, increased healthcare costs, and overall treatment inefficacy. In developing countries like Pakistan, where the healthcare system faces numerous challenges, the prevalence of such errors is higher [12–15]. Various cross-sectional studies in Pakistan have assessed the knowledge and awareness of drug prescriptions among dental practitioners and house officers. For instance, Ashraf et al. [12]. found that 50.5% of respondents were unfamiliar with WHO guidelines for good prescribing. Similarly, Baig et al. [13]. reported inadequate compliance with these guidelines. Ashraf et al. [14] noted that the majority of house officers relied on their supervisors for drug prescriptions, with the Internet (37%), books (35.8%), and fellow colleagues (34.3%) being other major sources of information. Babar et al. [15]. highlighted a general lack of knowledge among dental house officers regarding prescription writing, as they were unaware of the essential elements of a prescription. While these studies were undertaken using questionnaires to assess knowledge and awareness, our study uniquely evaluates real-time prescriptions to provide a more accurate assessment of current prescribing practices among junior dental practitioners.

Moreover, evaluating the quality of prescriptions written by junior dental practitioners in a tertiary care hospital in Karachi provides valuable insights into common errors and areas needing improvement. This study aims to identify the frequency and types of prescription errors, assess adherence to WHO prescribing indicators [8], and highlight the gaps in current prescribing practices. By doing so, it seeks to inform targeted interventions, such as enhanced training programs and stricter regulatory measures, to improve the quality of prescriptions, thereby ensuring better patient outcomes and minimizing the risks associated with incorrect drug prescriptions.

## Materials and methods

### Study setting and ethical approval

This cross-sectional study was conducted from January 2021 to March 2021, during which verbal informed consent was obtained from the participants whose prescriptions were evaluated. Given that the study involved the analysis of existing medical records and did not include direct interaction with participants or patients, verbal consent was considered appropriate and was approved by the Institutional Review Board/Ethics Committee. Prior approval was obtained from the ethics and review committee of the tertiary care hospital, JPMC: No. F.2–81/2020-GENL/12,316/JPMC. Participants were fully informed about the nature and purpose of the study before providing consent.

### Study selection criteria and sample estimation

The sample size was calculated using the WHO [16] sample size calculator. Considering the average number of drugs per encounter is 37.9% or 3.2 [21], with a confidence interval of 95%, a margin of error of 5%, and a power of test of 80%, and incorporating an anticipated response rate of 75% and an estimated effect size of 0.5, the projected sample size for this study was 466 participants. This study employed non-probability convenience sampling method. This approach involved including all prescriptions written by house surgeons and postgraduate medical trainees for walk-in patients visiting the dental outpatient department. We incorporated an anticipated response rate of 75% and an estimated effect size of 0.5. With these considerations, alongside a 95% confidence interval, a 5% margin of error, and 80% power, the revised sample size for this study was 466 participants.

### Prescribing indicators

The WHO core drug prescribing indicators [8] are a set of measures designed to assess the quality of prescribing practices by healthcare professionals. These indicators are commonly used in healthcare research to evaluate the appropriateness and efficiency of drug prescribing. It includes the average number of drugs per encounter, the percentage of drugs prescribed by generic name, the percentage of encounters with antibiotics prescribed, and the percentage of drugs prescribed from the essential drug list.

#### Average number of drugs per encounter

This indicator assesses the extent to which healthcare practitioners prescribe multiple medications during a single patient encounter. It is calculated by dividing the total number of drugs prescribed by the total number of patient encounters. A high average number of drugs per encounter may suggest polypharmacy, which can increase the risk of adverse drug interactions and medication non-adherence.

#### Percentage of drugs prescribed by generic name

This indicator measures the proportion of drugs prescribed using their generic names rather than brand names. A higher percentage of generic drug prescribing is generally considered more cost-effective and less prone to medication errors.

#### Percentage of encounters with antibiotics prescribed

This indicator evaluates the frequency with which antibiotics are prescribed during patient encounters. It is calculated by dividing the number of encounters with antibiotics prescribed by the total number of patient encounters. Overuse or inappropriate use of antibiotics can contribute to antibiotic resistance, a global health concern.

#### Percentage of drugs prescribed from the essential drug list

This indicator assesses the extent to which prescribed medications are included in an essential drug list, which typically comprises a limited number of cost-effective and therapeutically effective drugs recommended for common health conditions. It is calculated by dividing the number of drugs prescribed from the essential drug list by the total number of drugs prescribed.

### Prescription errors

The prescription error parameters were prepared by studying WHO practical manual on guide to good prescribing [7] and previous studies [3, 4, 10, 11, 13]. The prescription errors including the errors of omission related to the physician (name, address, medical license number, specialty, contact details, signature, diagnosis, date of prescription) and the patients (name, age, weight, gender address) were evaluated. Moreover, errors of omission related to the missing information about the drug was also noted (drug generic name, drug form, drug strength, frequency, route of the drug, duration of treatment, and the number of the drugs). Furthermore, the errors of commission (wrong drug, wrong dose, wrong dosage form, wrong strength, possible drug-drug interaction) were noted. The consultants determined whether a drug prescription was incorrect by assessing if the prescribed medication aligned with the diagnosed condition and adhered to accepted medical standards and guidelines.

Lastly, the layout of the prescription was evaluated. Sign of the treatment and illegible handwriting was noted.

### Statistical analysis

The statistical analysis was carried out with SPSS version-25. The descriptive analysis was done for qualitative variables (age and gender indication, drugs/encounters, number of drugs per encounter, and various drugs prescription) to calculate frequency and percentages.

## Results

### Prescribing indicators

The WHO prescribing indicators including the average number of drugs per encounter, the percentage of drugs prescribed by generic name, the percentage of encounters with antibiotics prescribed, the percentage of encounters with injection and the percentage of drugs prescribed from the essential drug list are presented in Table 1.

**Table 1:** Prescribing indicators (*n* = 466)

s. no.	Prescribing Indicators assessed	Total drugs/encounters	Average/ Percentage	WHO standard derived or ideal (%)
1	Average number of drugs per encounter	1575	3.378	1.6–1.8
2	Percentage of encounters with antibiotics	452	96.99%	20–26.8%
3	Percentage of encounters with an injection	10	2.14%	13.4–24.1%
4	Percentage of drugs prescribed by generic	79	16.95%	100%
5	Percentage of drugs from the essential drug list	371	23.55%	100%

*Prescribing Indicators assessed: This column lists the different metrics used to evaluate prescribing practices. each row represents a specific indicator. **Total drugs/encounters: It indicates how many times a particular action (such as prescribing drugs) occurred across all encounters. ***Average/Percentage: This column displays either the average number of drugs per encounter or the percentage of encounters involving a specific action ****WHO standard derived or ideal (%): This column provides the ideal percentage or range according to WHO standards for each prescribing indicator. It serves as a benchmark to compare the actual prescribing practices

### Prescription errors (*n* = 466)

The prescription errors found in the selected prescriptions were evaluated and are presented in Table 2.

#### **Omission errors related to the physician**

Out of the 466 prescriptions, the name of the physician was mentioned in 275 (59%) and was missing on 191 (40.9%) prescriptions, whereas the address and specialty of the prescriber were missing in the majority, 452 (97%) of the prescriptions. Most of the prescriptions also lacked contact details of the prescriber 419 (89.9%) and date 261 (56%). Moreover, none of the prescribers mentioned their medical license number, 466 (100%). Only a small number of prescriptions had a signature missing on them, 35 (7.5%). Lastly, the majority of the prescriptions lacked diagnosis, 409 (87.8%) and had illegible handwriting 299 (64%) as depicted in Table 2.

#### **Omission errors related to the patient**

The majority of the prescriptions lacked patient details, including, age and address were missed in 453 (97.2%) whereas none mentioned the weight of the patient as depicted in Table 2.

#### **Omission errors related to the drug**

The drug-related errors include missing form and route of drug 14 (3%), missing drug strength 69 (14.8%), missing frequency 126 (27%) and duration of treatment 72 (15.4%). Ironically, 442 (94.8%) prescribers failed to use the drug’s generic name.

#### **Commission errors**

The errors of the commission were comparatively fewer but were still noteworthy. Wrong drug dosage was prescribed by 89 (19%) prescribers followed by the wrong drug in 52 (11.1%), wrong strength 43 (9.2%) and wrong form in 9 (1.9%). Out of 1575 medicines prescribed in 466 prescriptions, 426 (27.04%) drug interactions were found.

**Table 2:** Prescription errors (*n* = 466)

s. no	Types of prescription errors		Number of errors *n* (%)
1.	**Omission errors related to the physician**	Name	191 (40.9%)
		Address	452 (97%)
		Medical License No.	466 (100%)
		Specialty	452 (97%)
		Contact Details	419 (89.9%)
		Date Of Prescription	261 (56%)
		Diagnosis	409 (87.8%)
		Signature	35 (7.5%)
2.	**Omission errors related to the patient**	Name	414 (88.8%)
		Age	453 (97.2%)
		Gender	14 (3%)
		Weight	466 (100%)
		Address	453 (97.2%)
3.	**Omission errors related to the medicines**	Drug Generic Name	442 (94.8%)
		Drug Form	14 (3%)
		Drug Strength	69 (14.8%)
		Frequency	126 (27%)
		Route	14 (3%)
		Duration Of Treatment	72 (15.4%)
**4.**	**Errors of commission**	Wrong Drug	52 (11.1%)
		Wrong Form	9 (1.9%)
		Wrong Strength	43 (9.2%)
		Wrong Dosage	89 (19%)
		Possible Drug-Drug Interaction	426 (27.04%)

### **Pattern of drug prescription**

**Fig. 1:**
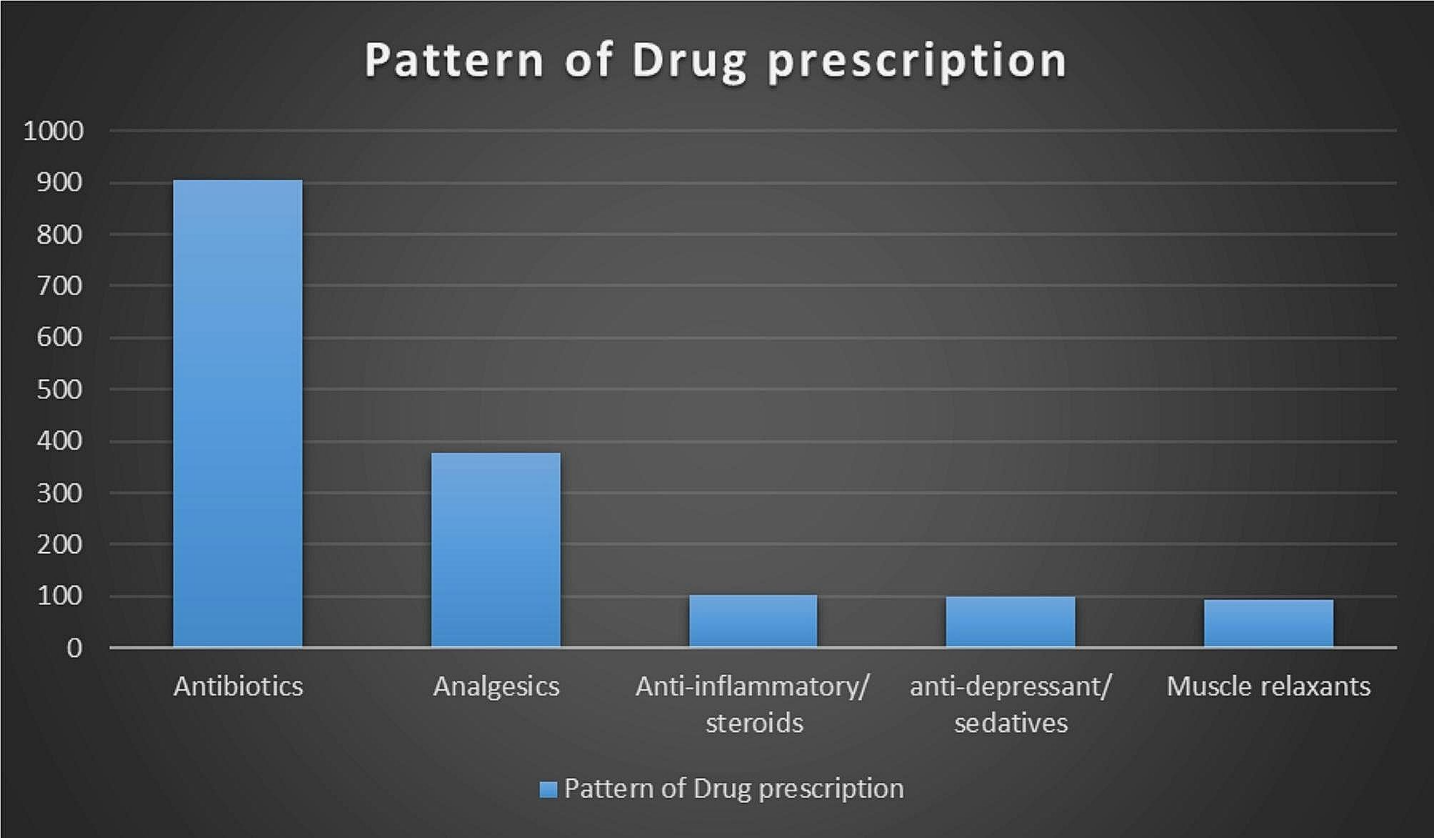
The pattern of drugs prescription

Out of the total, 466 prescriptions, 1575 drugs were prescribed with an average of 3.378 drugs per prescription. A large number of antibiotics, 904 (57.3%) were prescribed followed by analgesics, 377 (23.9%), 102 (6.47%) anti-inflammatory/steroids, 98 (6.2%) anti-depressant/ sedatives and 94 (5.96%) muscle relaxants as presented in Fig. 1.

### Layout of the prescription

About 353 (75.7%) participants wrote down the sign of the treatment on the prescription paper. Furthermore, illegible handwriting was observed in 151 (32.4%) prescriptions.

## Discussion

Undesirable clinical outcomes may result due to inappropriate prescribing practices, which is an important threat to patient safety. Implementation of appropriate prescribing skills in community settings, where the majority of prescriptions are written, offers a critical area of opportunity to improve the quality of care and treatment outcomes [7]. Therefore, this study was undertaken to evaluate if the prescriptions are following WHO core prescribing indicators and compare the practitioner’s prescription with the prescribing guidelines of World Health Organization.

The average number of drugs per encounter according to WHO should be 1.6–1.8 [8], whereas in our study a total of 1575 drugs were prescribed at an average of 3.378 drugs per prescription which is quite high. The results are in contrast with a study by Wendie et al., where 3199 drugs were prescribed with an average number of 2.1 drugs per prescription and antibiotics were prescribed in 660 (43.9%) [21] encounters while in our study 452 antibiotics (96.99%) were prescribed. The frequency of antibiotics prescribing was extremely high than the WHO standard (20–26.8%) [7]. Our results are in accordance with Shrestha et al., who also observed low compliance with WHO prescribing indicators in their study where 2448 drugs were prescribed at an average of 3.2 but the frequency of antibiotics prescription was far less 292 (37.9%) [11]. Polypharmacy or irrational antibiotic use could be due to a lack of therapeutic knowledge or a lack of clinical practice guidelines. Thompson et al. [17] and Minallah et al. [18], also concluded that overuse and misuse of antibiotics in dentistry can contribute to the development of antibiotic resistance. Specifically, unnecessary antibiotic prescriptions for dental procedures such as extractions and root canals can lead to the emergence of antibiotic-resistant bacteria in the oral cavity. These bacteria can then spread to other parts of the body or to other individuals, leading to more difficult-to-treat infections. Therefore, it is important for dental professionals to prescribe antibiotics only when necessary and to follow appropriate prescribing guidelines to help prevent the development of antibiotic resistance. They also suggested implementing evidence-based prescribing guidelines, improving communication with patients, and using alternative therapies when appropriate. Therefore, low drug prescribing and rational antibiotic use should be strictly practiced and monitored as it can reduce the chances of drug interaction, unwanted adverse effects, bacterial resistance and healthcare costs [19, 20].

Drug prescribing using a generic name rationalizes drug therapy and minimizes the cost of treatment. WHO recommends prescribing all drugs by their generic name except if there is a particular reason to prescribe a special brand but, in this study, it was observed that only 16.95% of drugs were prescribed by their generic names. The National Drug Policy (NDP) of Pakistan also indicates prescribing drugs by their generic names. Shrestha et al. [11], observed even much poorer generic prescribing of the drugs (2.9%) [11]. On the contrary much better results were observed in other studies by Wendie et al. [21], Akl et al. [22] and Sisay et al. [23] where drugs prescribing by the generic names were observed by 98%, 95.4% and 90.61% respectively. This difference could be since in Pakistan pharmaceutical representatives influence the prescribing pattern significantly and they are biased toward brand name medicine, which creates a negative attitude toward generic prescribing [24]. Furthermore, only 23.55% of drugs were prescribed from the essential drug list which is in accordance with Shrestha et al. [11] (21.3%) but in contrast to other studies by Wendie et al. [21] and Akl et al. [22] where drugs prescribed from the EDL were observed by 100% and 95.4% respectively. The reason could again be similar, i.e. promotion by pharmaceutical companies or lack of knowledge. Drug prescribing from the essential drug list should strictly be practiced and monitored as these medicines are cost-effective, qualitative and safe.

Studies suggest that the risk of iatrogenic damage resulting from wrong use of an ever-growing number of drugs (errors of commission) has increased, so has the potential of morbidity and mortality from inadequate treatment (errors of omission) [10, 11, 13, 19, 22]. In this study, the errors of omission related to the physicians were very high. The majority skipped all the major details including 40.9% did not mention their names, 97% missing their address, 89.9% missing their contact details and none of them mentioning their license number which is in accordance to a study by Phalke et al. [25], where the contact details and registration number of the doctor were found missing in about three fourth of the prescriptions. Singh et al. [26] also observed similar findings, where 100% missed providing the contact details and only 3.3% provided their license number. Similarly in a study by Irshaid et al. [27], none of the prescriptions included the contact details of the prescriber although only 16.7% failed to miss their names. Singh et al. [26] and Irshaid et al. [27] mentioned that in their cases the contact details were not relevant as the physician can be reached through the telephone directory or the hospital pager system because their pharmacy department fills prescriptions coming from within the hospital. However, these reasons cannot justify the fact that these missing details were a violation of WHO guidelines. Moreover, the practitioner’s contact number or address is important so that they can be contacted in case of any emergency. Not mentioning the qualification or registration number on the prescription can raise questions about their authority to prescribe medicine. The pharmacist also uses these details to authenticate the prescription before dispensing whether the prescription is genuine or from a quack/ homeopathic or ayurvedic doctor. Furthermore, it is also particularly important in challenging the prescription in court [26–28].

Moreover, in this study, 56.1% failed to mention the date on the prescription and only 32.4% had illegible handwriting. Comparable results were observed by Irshaid et al. [27], where 56% of practitioners did not date the prescription. While Singh et al. [26] found contrasting results, where 98.3% of practitioners mentioned date and 95.8% had legible handwriting. Annually, around 7,000 mortalities have been reported due to medication errors with practitioner’s poor handwriting being the leading cause of medication dispensing errors [27, 28]. Such errors can be controlled by self- and external assessment, the use of technology including the prescription chart, information transfer between primary and secondary care, and the use of computerized prescribing and clinical decision support [29, 30]. The diagnosis of the patient was not mentioned by 87.8% of the patients in this study which is contrary to other studies including, Shrestha et al. and Singh et al., where 39% and 35.8% failed to mention the diagnosis respectively [11, 26]. Diagnosis information on prescriptions could help pharmacists identify safety issues. For handwritten prescriptions, it could prevent rare dispensing errors where illegibility causes a pharmacist to confuse one drug with another [31].

The errors of omission related to the patients were also extremely high as the majority failed to mention the age (97.2%) or address of the patient and none of them mentioned the weight of the patient. About 88.8% failed to mention the name of the patient as well. These findings are similar to a study by Sheikh et al. [28], in which 78.2% of prescriptions did not have patient’s age and none had the patient weight or address. Obaid et al. [32], found contrasting results, where 82.2% had mentioned patient age. But similar to our findings, the patient’s weight was missing on 98.9% of prescriptions which is especially important for deciding the dose of the medication. Similarly, none of them mentioned the address of the patient. Correct patient detail is important for medicolegal purpose, and it also ensures patient’s safety. The clear details of a patient’s age, weight and most importantly the diagnosis helps the pharmacist to identify and confirm the medicine dose and dosage form to dispense especially when these are not legible in the prescription and communication with the prescriber is not possible [29].

Regarding the omission errors related to drugs, the frequency of the drug was missed by 27%, followed by duration 15.4%, drug strength 14.8%, drug form and route 3% by the prescribers in this study. Comparable results were observed by Gul et al. [33], where strength was missed by 16.7%, followed by drug form 13.3%, route 11%, duration 7.65% and frequency 4.3%. Moreover, the errors of the commission were comparatively less but were still noteworthy. Wrong drug dosage was prescribed by 19% of prescribers followed by wrong drug in 11.1%, wrong strength 9.2% and wrong form 1.9%. Comparable results were observed by Gul et al. [33], where wrong strength was prescribed by 28% followed by wrong dose 17%, wrong form 14.6%, wrong drug 7.8% and wrong route 5.6% respectively. On the contrary, even better results were observed in a study by Thirumagal et al. [34], where wrong frequency was observed in 10.3% of prescriptions followed by wrong dose 0.5%, wrong route 0.3% and wrong dosage form 0.3%. The most obvious reason for this kind of error could be the lack of clinical knowledge. Hence, these findings indicate the need to introduce the concept of medication safety as early as the undergraduate level. Physicians need to be trained to make decisions based on reliable and current evidence.

The external validity of our study, conducted among junior dental practitioners in a tertiary care hospital was influenced by factors such as the study’s specific healthcare setting, cultural and regulatory context, and the study’s time frame. While our findings may not be directly generalizable to all dental practitioners in diverse settings, they underscore the need for education, training, and regulatory oversight to improve prescription practices, emphasizing adherence to WHO prescribing guidelines, rational drug use, and medication safety. Moreover, it is suggested that the administrative monitoring of the prescription habits of physicians, especially junior doctors should be carried out. Furthermore, continuous professional educational programs for doctors, pharmacists, and technicians should be undertaken as they could improve the quality of prescription writing as well as the proficiency of prescription screening before dispensing medications to the patients.

### Limitations of the study

The study was conducted in a single department of a tertiary care hospital in Karachi, Pakistan, which may not be representative of prescription writing practices in other departments of the same hospitals or other healthcare settings. Moreover, in our study, we focused specifically on evaluating the quality of prescriptions written by junior dental practitioners, which is why the encounters resulting in a drug prescription were only included rest were excluded. The study did not assess the reasons behind the prescription errors, such as lack of knowledge, time pressure, or inadequate training, which could have provided insights into addressing the prescription writing issues.

## Conclusion

The study revealed that the prescription writing practices among junior dental practitioners are below optimum standards. The average number of drugs per encounter was 3.378, which is significantly higher than the WHO standard of 1.6–1.8. A substantial percentage (96.99%) of encounters involved antibiotics, which far exceeds the WHO recommended range of 20–26.8%. However, only 16.95% of drugs were prescribed by generic name, and 23.55% were from the essential drug list, both of which fall short of the ideal 100% standard. Numerous prescription errors, both omissions and commissions, were identified, highlighting the need for improved training and adherence to WHO guidelines on good prescribing practices. Implementing targeted educational programs and stricter regulatory measures could enhance the quality of prescriptions and overall patient safety.

## Data Availability

The data included in the present study are available upon request from the corresponding author.
